# Protein abundances can distinguish between naturally-occurring and laboratory strains of *Yersinia pestis*, the causative agent of plague

**DOI:** 10.1371/journal.pone.0183478

**Published:** 2017-08-30

**Authors:** Eric D. Merkley, Landon H. Sego, Andy Lin, Owen P. Leiser, Brooke L. Deatherage Kaiser, Joshua N. Adkins, Paul S. Keim, David M. Wagner, Helen W. Kreuzer

**Affiliations:** 1 Chemical and Biological Signature Sciences, Pacific Northwest National Laboratory, Richland, Washington, United States of America; 2 Applied Statistics and Computational Modeling, Pacific Northwest National Laboratory, Richland, Washington, United States of America; 3 Department of Genome Sciences, University of Washington, Seattle, Washington, United States of America; 4 Pathogen and Microbiome Institute, Northern Arizona University, Flagstaff, Arizona, United States of America; 5 Biological Sciences Division, Pacific Northwest National Laboratory, Richland, Washington, United States of America; George Mason University, UNITED STATES

## Abstract

The rapid pace of bacterial evolution enables organisms to adapt to the laboratory environment with repeated passage and thus diverge from naturally-occurring environmental (“wild”) strains. Distinguishing wild and laboratory strains is clearly important for biodefense and bioforensics; however, DNA sequence data alone has thus far not provided a clear signature, perhaps due to lack of understanding of how diverse genome changes lead to convergent phenotypes, difficulty in detecting certain types of mutations, or perhaps because some adaptive modifications are epigenetic. Monitoring protein abundance, a molecular measure of phenotype, can overcome some of these difficulties. We have assembled a collection of *Yersinia pestis* proteomics datasets from our own published and unpublished work, and from a proteomics data archive, and demonstrated that protein abundance data can clearly distinguish laboratory-adapted from wild. We developed a lasso logistic regression classifier that uses binary (presence/absence) or quantitative protein abundance measures to predict whether a sample is laboratory-adapted or wild that proved to be ~98% accurate, as judged by replicated 10-fold cross-validation. Protein features selected by the classifier accord well with our previous study of laboratory adaptation in *Y*. *pestis*. The input data was derived from a variety of unrelated experiments and contained significant confounding variables. We show that the classifier is robust with respect to these variables. The methodology is able to discover signatures for laboratory facility and culture medium that are largely independent of the signature of laboratory adaptation. Going beyond our previous laboratory evolution study, this work suggests that proteomic differences between laboratory-adapted and wild *Y*. *pestis* are general, potentially pointing to a process that could apply to other species as well. Additionally, we show that proteomics datasets (even archived data collected for different purposes) contain the information necessary to distinguish wild and laboratory samples. This work has clear applications in biomarker detection as well as biodefense.

## Introduction

The techniques collectively known as “omics” are in effect massively multiplexed biological measurement technologies, able to measure hundreds or thousands of genes, proteins, or metabolites simultaneously. These technologies have proven to be highly effective tools for probing the effects of environmental perturbations and understanding the regulation of biological systems [1–6]. In fact, the output of omics measurements can be used as a measure of the internal state of a biological system. DNA sequence data has revolutionized our understanding in many areas of biological science, largely because of the existence of public data repositories that can be mined and the development of software for genome sequence analysis. Fully exploiting the flood of information-rich data from omics techniques requires matching advances in statistical methods, and the development of approaches for exploiting growing public archives of omics data.

Here we demonstrate the application of one statistical approach, the lasso logistic regression classifier [7, 8] to data from one omics technology, liquid chromatography-tandem mass spectrometry (LC-MS/MS) proteomics, to characterize the origin of *Yersinia pestis* samples as laboratory-adapted or environmental strains. The proteomics data included data from our laboratory as well as data mined from an archive that had been generated from samples produced by different researchers over several years. We show that the lasso logistic regression method can produce a robust and highly accurate classifier from proteomics data, even when data acquisition and sample preparation are not carefully controlled as in a traditional proteomics experiment. Our results suggest a new application for sample classification using protein expression data. In addition our results suggest that, given the appropriate metadata, the methodology could allow retrospective analyses or meta-analyses of factors not targeted in the original study design. Our work highlights the importance of data sharing (including sample metadata) and the usefulness of public data repositories [9, 10]. This study is not a classic biomarker discovery or validation effort; it does not represent a validation/verification of any particular set of protein signatures. It does demonstrate that adaptation to laboratory conditions involves broad changes in protein expression that can be used to classify samples as originating from wild or laboratory strains. Further, we show that the same data can be used to classify samples with respect to other characteristics, such as growth medium, highlighting the potential richness of information that can be derived from proteomics.

### Background

The environment a microbe experiences during cultivation in a laboratory is very different from its natural environment. Laboratory cultures are temperature-controlled, commonly nutrient-rich, and free from other hostile conditions such as host immune factors and competing organisms. Bacteria can adapt quickly to these conditions, both by altering gene expression and through adaptive evolution, as has been illustrated through the work of Lenski and his colleagues, who have extensively studied how a strain derived from the laboratory strain *E*. *coli* B evolves over time under various laboratory conditions (e.g., [11]). Over time, and with repeated culturing, including sharing between laboratories and researchers, laboratory strains diverge in genotype and phenotype from their wild ancestors. Such divergence could have important implications for the relevance of research performed with laboratory-adapted strains.

We recently isolated *Y*. *pestis* from fleas collected in two distinct regions of the United States and serially passaged multiple sub-cultures of each isolate in a standard rich medium commonly used to propagate *Y*. *pestis* in the laboratory. We then compared whole genome sequences, proteomes, and carbohydrate (monosaccharide) profiles of the ancestor isolates and their laboratory-adapted descendants in an effort to understand how the organism changes during prolonged laboratory culture [12]. We discovered that there were consistent changes in protein expression among the passaged descendant strains that, surprisingly, were *not* always obviously associated with underlying genomic changes [13].

These protein abundance changes were evident even though both the wild isolates and their descendants had been cultured in the same laboratory growth medium prior to analysis. The wild isolates had in fact been subjected to three culture steps in the process of isolating them from the fleas, confirming identity and purity, and producing biomass for analysis. The protein expression changes observed in the passaged descendant strains thus appeared to be adaptive changes engendered by more prolonged laboratory cultivation, and not immediate responses to the specific growth environment. Preliminary examination of *Y*. *pestis* proteomic data from a few un-related experiments using two standard laboratory strains, the avirulent KIMD27 [14] and the virulent CO92 [15], suggested that protein abundance differences might provide a general signature of laboratory adaptation.

We therefore took advantage of the archived data at the proteomics facility located at the WR Wiley Environmental and Molecular Sciences Laboratory (EMSL) at the Pacific Northwest National Laboratory (PNNL), which has, over a period of years, performed mass spectrometry-based proteomic analysis for numerous studies, addressing diverse biological questions and targeting hundreds of different organisms [13, 16]. At this writing, the archive contains over 508,000 LC-MS datasets collected on diverse samples. We extracted all *Y*. *pestis* proteomics data collected on non-fractionated samples from the EMSL archive, combined it with our data from the previous study as well as from our own unrelated proteomic studies of *Y*. *pestis*, and tested whether proteomics data could differentiate wild isolates from the laboratory strains.

We show that despite different genetic strains, growth media, growth temperatures, growth stages, sample preparation procedures, and analytical instruments, protein abundance data could reliably differentiate wild isolates from laboratory-adapted strains. This result suggests that *Y*. *pestis* adapts to long-term laboratory culture by altering patterns of protein expression in predictable ways. In addition to pointing towards a direct signature of laboratory adaptation in *Y*. *pestis*, our work also suggests that proteomic data (both protein identification and abundance data) from unrelated experiments and facilities can be combined and mined to garner useful information.

## Materials and methods

### Bacterial samples

All growth and processing of live *Y*. *pestis* was carried out in the certified BSL3 facility at Northern Arizona University. Wild *Y*. *pestis* strains were isolated from fleas collected from black-tailed prairie dog (*Cynomys ludovicianus*) colonies and from Gunnison’s prairie dog colonies (*C*. *gunnisoni*) showing signs of recent die-off’s as previously described [17, 18]. Because fleas were collected from burrows after rodent hosts had already died, no prairie dogs were harmed or killed for the purpose of collection. Collection of Yp1945 and Yp2126 is described in [12]. Fleas were pooled by burrow and homogenized in BHI broth supplemented with 10% glycerol. The homogenized suspensions were plated onto cefsulodin, irgasan, and novobiocin (CIN) agar plates and incubated at 28°C for 48 h. Suspected *Y*. *pestis* colonies were purified onto sheep blood agar, and their identity confirmed by a real-time PCR-based assay targeting the plasmid-borne *pla* gene [25, 26]. Confirmed *Y*. *pestis* isolates were spread onto a fresh sheep blood agar plate. Wild *Y*. *pestis* isolates used were passaged no more than three times during the isolation process. Wild *Y*. *pestis* strains were cultured in 15 ml BHI broth, tryptic soy broth, or LB broth at 28°C for 48 h (i.e. into stationary phase) in 50 ml conical tubes.

### Liquid chromatography-tandem mass spectrometry data analysis

*Y*. *pestis d*atasets generated for this endeavor and archived *Y*. *pestis* datasets from two administratively distinct and physically separated research groups (essentially two different laboratories) at PNNL were pooled together. The resulting set of 381 datasets originated from a wide range of experiments conducted over the course of several years. Protein extracted from all the samples had been digested with trypsin and analyzed by liquid chromatography-tandem mass spectrometry. For this study, the raw mass spectrometry files were re-analyzed with the program MaxQuant [[19], version 1.5.1.2] to identify and quantify proteins present, and compiled into a matrix where each line represented a protein detected in at least one sample, each column represented one dataset, and each cell contained the abundance of the respective protein in the respective dataset. Details of the data processing are given in Data Processing in the Supplemental Information, and sample and dataset metadata are presented in S1 Dataset. The mass spectrometry proteomics data have been deposited to the ProteomeXchange Consortium via the PRIDE [55] partner repository with the dataset identifiers PXD007254, PXD002955 and PXD002961. (Note that PXD002955 and PXD002961 are the datasets previously described in reference [12], which have been incorporated into this work).

### Logistic regression classifier

The LRC method is computationally intensive and uses an iterative process to identify a relatively small number of features that can be used to accurately predict a binary outcome, such as whether a sample contains a wild or laboratory strain. The output of the LRC includes a list of the features (here, proteins) selected for their ability to discriminate the two classes of samples along with their corresponding coefficients in the logistic regression model. The LRC also utilizes two tuning parameters, *λ* and *τ*. The value of *λ* determines weight of the Lasso penalty, where larger values of *λ* lead to fewer features with smaller logistic regression coefficients and smaller values of *λ* lead to more features with larger coefficients. The value of *τ* is a threshold such that if the probability that a sample is a laboratory strain (as predicted by the logistic regression model) exceeds *τ*, the sample is classified as being a “laboratory” strain. If the predicted probability is less than or equal to *τ*, the sample is classified as being a “wild” strain. The values of *λ* and *τ* are chosen to minimize the classification error estimated via cross-validation.

Cross-validation involves randomly partitioning the data into, in this case, 10 non-overlapping groups, or folds. Nine of the folds (90% of the data) are combined to form a dataset used to train the logistic regression model which is then tested by predicting the strains of the samples in the remaining fold (10% of the data). The process is repeated so that ten logistic regression models (trained from the 10 possible training sets) are tested by predicting the corresponding ten test sets. This process provides an estimate of the classification error for a specific value of *λ* and *τ*. Using the same random partition, the cross-validation process is repeated for multiple values of *λ* and *τ* until the values of *λ* and *τ* that minimize the classification error are identified. The classification error is calculated as the number of misclassified samples divided by the total number of samples that are classified. No distinction was made between false positive and false negative errors when calculating the classification error.

To ensure the process is robust to the particular random partition of the data, the entire process is repeated (in what is called a cross-validation replicate) for other random partitions of the data, each yielding “optimal” values of *λ* and *τ*, and an estimate of the classification error. In this study, we performed 100 cross-validation replicates. The median of the 100 “optimal” values of *λ* and the median of 100 “optimal” values *τ* of provided the overall optimal estimates of *λ* and *τ*. These overall optimal estimates were then used to refit the logistic regression models using the same random partitions and replicated cross-validation process to obtain an overall estimate of the classification error, which was the average of the 100 estimates of the classification error observed from each cross validation replicate. The accuracy values we report in the Results are the overall estimates of the classification error subtracted from one.

The final LRC is given by fitting the logistic regression model to all the samples using the overall estimates of *λ* and *τ*. The protein features selected in this final LRC (and their corresponding coefficients) are signatures that distinguish the wild and laboratory strains. This final LRC is the model we would use to predict the strain of future samples. A mathematical treatment of the LRC method is provided in the Supplemental Information.

### Data availability

A comprehensive description of all mass spectrometry datasets used in this study can be found in supplemental Dataset S1. All data used in this paper has been deposited to the PRIDE proteomics data repository under accession numbers (to be submitted after the manuscript has been provisionally accepted) for more convenient retrieval.

## Results

### The samples

A summary of the samples represented in our dataset is presented in Table 1. Our previous serial passage study [12] involved only two different wild isolates cultured in a single laboratory medium (brain-heart infusion broth). These two wild isolates generated 11 and 12 independent lineages, respectively, that were serially passaged 60 times, or ~750 generations. Whole-genome sequence analysis showed that the 23 passaged lineages had diverged genetically over the course of the passaging; thus, they represent genetically distinct laboratory-adapted strains. Our broader research group also had published [20] and unpublished proteomic data from unrelated studies using the avirulent *Y*. *pestis* laboratory strain KIMD27 [14]. The data in the EMSL archive, gathered from experiments by various research efforts and collaborations [[21, 22] and unpublished data] was derived from the widely-used virulent North American laboratory strain CO92 [15] and three mutant derivatives of CO92. The virulent *Y*. *pestis* cultures were produced in biosafety level 3 (BSL3) containment facilities at other institutions, inactivated, and then sent to PNNL for analysis. For the current study, we cultured an additional 8 geographically diverse U.S. *Y*. *pestis* isolates in the certified BSL3 facility at Northern Arizona University (NAU), using up to three different media per isolate (a few of the isolates did not grow in one of the media). We also grew additional cultures of the two isolates used in our serial passaging study [12] in additional media.

**Table 1:** Overview of the samples represented in the assembled data sets.

	Description	Number of genotypes	Number of cultures	Growth medium	Growth temps, °C	Time of culture	Inactivation methods	Instruments and facility
Wild isolates	Previous serial passaging	2	18	BHI*	28 or 29	48h	Ethanol treatment	Orbitrap LTQ in Facility 1
	Previous serial passaging	2	8	BHI	28 or 29	48h	Ethanol treatment	Orbitrap LTQ in Facility 2
	Additional isolates	8^Ϯ^	25	BHI, LB^ǂ^ or TSB^§^	28 or 29	48h	Ethanol treatment	Orbitrap LTQ in Facility 1
Laboratory strains	Previous serial passaging	16	16	BHI	28	48h	Ethanol treatment	Orbitrap LTQ in Facility 1
	Previous serial passaging	7	7	BHI	28	48h	Ethanol treatment	Orbitrap LTQ in Facility 2
	CO92 and derivatives	4	41	BCS^#^ or DMEM**	26 or 37	1,2,4,8h or other^¶^	8M urea	Orbitrap LTQ or Velos Orbitrap in Facility 2
	KIMD27	1	19	BHI	28 or 30	48h	Ethanol, autoclaving, or irradiation	Orbitrap LTQ in Facility 1
	KIMD27	1	3	BHI, LB or TSB	28	48h	Ethanol	Orbitrap in Facility 2

*Brain-heart infusion;

^Ϯ^Additional cultures of the wild isolates used in the serial passaging experiment were also grown in LB and TSB for this study; they are not included in the 8 genotypes but are included in the 25 cultures;

^ǂ^Luria-Bertani broth;

^§^Tryptic soy broth;

^¶^The time cultures were grown prior to harvest was not always provided in the archived data; some of the laboratory cultures may have been grown for different periods of time than those listed;

^#^Best case scenario medium [23];

**Dulbecco’s Modified Eagle Medium

Assembling all the data from our published and unpublished work, the new data from the additional wild isolates, and the EMSL data resulted in 381 proteomics datasets collected on 137 independent cultures of *Y*. *pestis*; most of these biological samples had been analyzed at least in triplicate. These samples were produced for diverse experiments studying effects of phenomena such as changes in growth medium, presence of antibiotics, temperature shifts, mutations, inactivation methods, and laboratory evolution on the *Y*. *pestis* proteome. The number of replicate cultures (see Table 1 and S1 Dataset) varied with the design of the original studies. Finally, because all of the wild samples were originally processed and analyzed in Facility 1, we re-processed and analyzed an aliquot of the remaining biomass from fifteen samples from the serial passaging study (originally conducted in Facility 1) in Facility 2 and used the Facility 2 data in the aggregated dataset (see Table 1). Altogether, 51 of the 137 samples were independent cultures of 10 different wild isolates and the remaining 86 were independent cultures of 28 laboratory-adapted strains. The range of growth and sample preparation conditions is summarized in Table 1; complete information on all samples is provided in S1 Dataset of the Supplemental Information.

It was our goal to determine whether proteomic data could be used to distinguish wild from laboratory strains despite all the potentially confounding factors in the sample sets. There were unequal numbers of laboratory and wild isolates in the sample sets, and unequal numbers of culture conditions and replication of various genotypes. The samples were produced under varying conditions that are well known to influence protein expression in bacteria in general and in *Y*. *pestis* in particular: growth medium, growth stage, and growth temperature [24–28]. Additional potential confounding factors included different procedures for inactivating the biomass prior to analysis, demonstrated to have a small but measurable effect on proteomic data [20], different cell lysis methods, differences in data collection, and the two different facilities in which samples were processed and analyzed. This aggregated dataset offered the opportunity to determine whether information can be gleaned from archived proteomic data that transcends the original intents of the disparate experiments represented in the dataset.

### The data

Proteomic analysis of all the samples was conducted by first extracting the proteins from cell mass, digesting them with trypsin, and then analyzing the digested peptides via liquid chromatography-tandem mass spectrometry. We assembled raw mass spectrometry data for every analysis of every sample and used standard methods of proteomic data analysis (see S1 Methods) to identify proteins represented by the peptides. We generated a list of proteins and abundances for every analytical dataset. We then generated a data table for the statistical analysis by compiling a list of every protein detected in any analysis. This procedure gave a total of 2,068 proteins detected in at least one condition, out of 4,065 annotated genes in the *Y*. *pestis* CO92 genome. This relatively low coverage, even with varied growth conditions, is presumably a product of the large range of protein expression levels, and the limitations of shotgun proteomics measurements. We summarized each protein’s presence in each individual biological sample (culture) in two ways. First, we compiled a presence/absence list. For every biological sample, if a protein was detected in *any* of the replicate analyses of that sample, it was scored as present in that sample. Second, we scored the relative abundance of each detected protein in each individual biological sample (intra-dataset normalization). If a protein was not detected in a given replicate analysis, it received a relative abundance score of 0. We normalized the protein abundances in an individual biological sample by first adding 1 and then taking the base-2 logarithm of all the abundance scores. The log-transformed abundances were then linearly scaled so that the smallest log-transformed abundance within a biological sample was mapped to 0 and the largest log-transformed abundance score in the biological sample was mapped to 1. For each protein in the biological sample, the average of the transformed abundances was calculated from the replicate analyses of the sample. The final data set consisted of the list of all proteins observed in the collected datasets, with an indicator of presence/absence (1 or 0) and an averaged relative abundance score (potentially from 0 to 1.0) for each of those proteins in each biological sample. This dual approach mirrors standard practice in comparative proteomics, where, in addition to proteins with measurable quantitative expression differences, proteins that are detected in only one experimental condition (and therefore ineligible for a quantitative comparison) are also considered to be potentially differentially regulated.

### Classifying wild versus laboratory-adapted samples

Using either the relative protein abundances or the presence/absence indicators as features, we developed logistic regression classifiers (LRCs) to distinguish between the wild and laboratory strains. We fit the logistic regression model using the Lasso technique [7, 8], which serves to simultaneously select features and restrict the size of the logistic regression coefficients. We employed 10-fold cross-validation [7], repeated 100 times with different random partitions of 90% of the data into a training set and 10% into a test set, to estimate tuning parameters in the LRC and to estimate the classification error (the fraction of samples we expect the LRC to predict incorrectly). The net effect of this approach was to select the set of features (proteins) that minimized the classification error. Additional details regarding the LRC method are provided in the Methods and the Supplementary Information.

**Fig 1:**
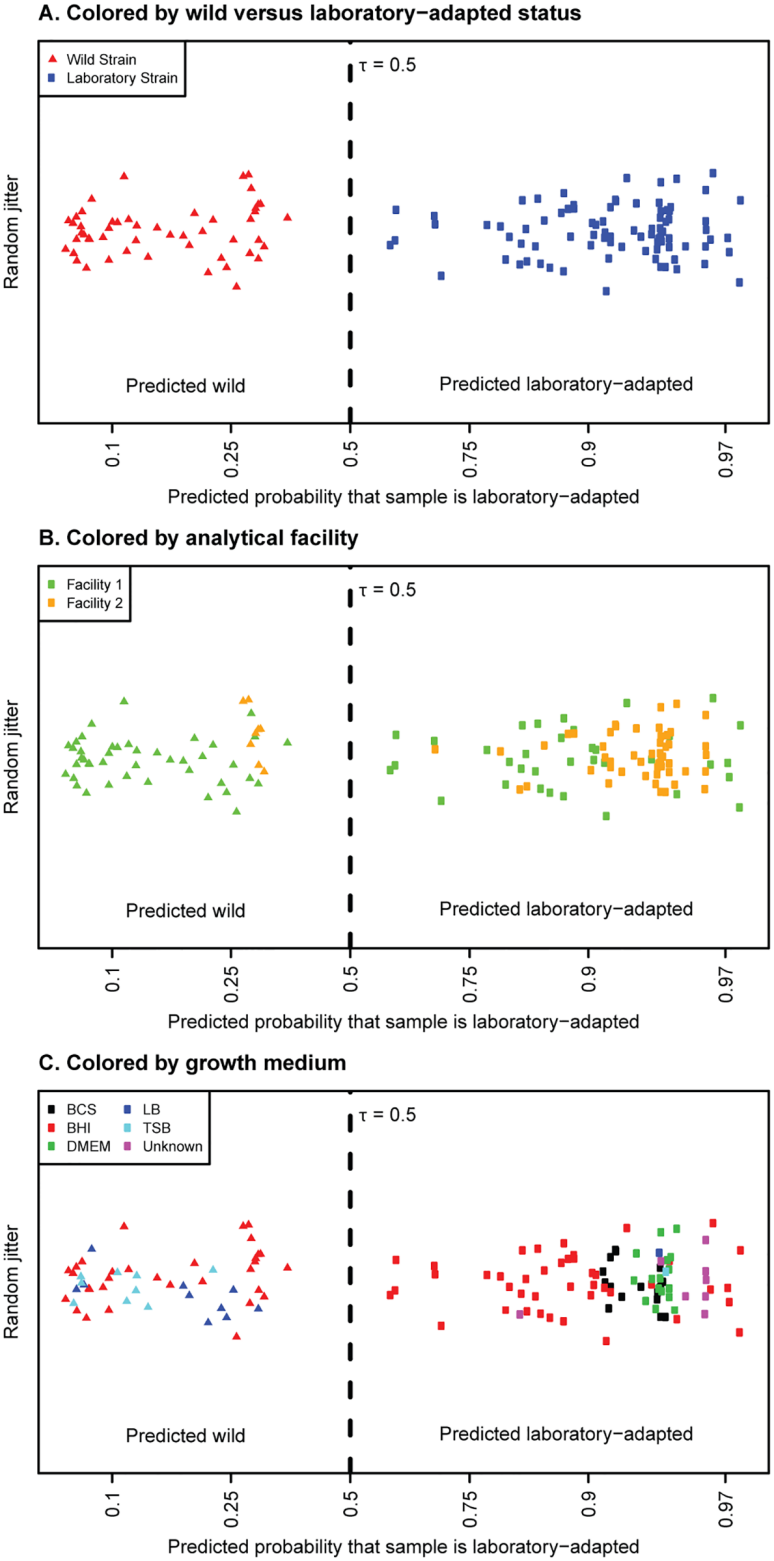
Output of the LRC to distinguish wild from laboratory-adapted strains using relative protein abundance data. Each symbol represents the prediction of the LRC for an independent culture. Triangles represent cultures of wild strains. Circles represent laboratory-adapted strains. The horizontal axis value is the predicted probability that a culture is laboratory adapted and is non-linear; points are separated vertically in a random fashion to improve the visualization. See Methods for an explanation of τ. A. Colors represent wild versus laboratory-adapted. B. Colors represent the facility of preparation and analysis. C. Colors represent the laboratory medium in which the cultures were grown prior to analysis.

The LRC built with relative abundances had an estimated accuracy (based on replicated 10-fold cross-validation; see Methods) of 99.5% (standard deviation 0.58%); the LRC built with presence/absence features had an estimated accuracy of 98.8% (standard deviation 0.59%). The output of the final LRC built with relative abundances is shown in Fig 1; the output of the classifier built with presence/absence data is shown in S1 Fig. The Lasso selected nine proteins for the final classifier when it was developed with the relative abundance data (Table 2) and 12 proteins for the classifier when it was developed with the presence/absence data (S1 Table). Both tables present the features arranged from that with the most positive coefficient to that with the most negative coefficient. For the presence/absence data, a positive coefficient indicates that the presence of the corresponding protein results in a higher probability the culture belongs to the laboratory strain. Conversely, a negative coefficient indicates that the presence of the protein results in a lower probability the culture belongs to the laboratory strain. For the relative protein abundances, the interpretation of the coefficients is similar: a positive (negative) coefficient indicates that, the larger the relative abundance of the corresponding protein, the more (less) likely the culture belongs to the laboratory strain.

**Table 2:** Protein features selected to distinguish wild and laboratory-adapted *Y*. *pestis* in the logistic regression classifier using relative protein abundance data.

Protein ^Ϯ^	Uniprot accession	LRC Coefficient*
Glucose-6-phosphate isomerase (EC 5.3.1.9)	Q8ZAS2	2.0398
Periplasmic thiol:disulfide interchange protein DsbA	Q9XBV2	1.5791
ATP synthase A chain (EC 3.6.3.14) ^ϮϮ^	Q7CFM3	0.6996
Periplasmic chorismate mutase I precursor (EC 5.4.99.5)	Q7CHH5	0.6561
Inorganic pyrophosphatase (EC 3.6.1.1)	Q8ZB98	0.1669
Maltose/maltodextrin ABC transporter, substrate binding periplasmic protein MalE	Q7CLD8	0.1122
Biofilm PGA synthesis deacetylase PgaB (EC.3-) (HmsF)	Q9R7V4	-0.4388
Attachment invasion locus protein precursor	Q0WCZ9	-0.9863
Sulfite reductase [NADPH] flavoprotein alpha-component (EC 1.8.1.2)	Q8ZBN6	-1.7647

*The final LRC also includes an intercept term of -0.0569 and optimal tuning parameters of *λ* = 0.0241 and *τ* = 0.5. See Methods.

^Ϯ^ Shaded cells indicate proteins whose abundance changed significantly between the ancestor wild isolates and the descendant lineages in our previous study [29]

^ϮϮ^ Although this polypeptide was not identified by Leiser et al [12] as significantly changing, the B subunit of the same protein was identified.

Interestingly, 4/9 of the features selected by the LRC trained with relative abundance data were proteins we had observed as significantly changing in abundance in our previous serial passaging experiment (shaded cells in Table 2; see Supplemental Spreadsheet 2 in [29]). These four features included the one with the most weight for classifying a strain as laboratory-adapted (glucose-6-phosphate isomerase) and the two with the most weight for classifying a strain as wild (Ail and sulfite reductase). The gene encoding the attachment/invasion locus (Ail) protein precursor was one of three hotspots for the accumulation of mutations during serial passage, but even in the lineages in which the gene maintained the wild-type Ail sequence, expression of the protein was greatly diminished compared to the ancestral strains (see Table 1 in [29]). Its negative coefficient indicates that increased abundance of Ail results in a higher probability the strain is wild. The fact that the Lasso selected features we had previously identified as significantly changing during our laboratory evolution experiment suggests that the LRC is selecting real features in the data that distinguish wild strains from their laboratory-adapted relatives.

The LRC also selected some relative abundance and presence/absence features that we did not observe to be significantly changing in the serial passage experiment, likely reflecting the inclusion of the many additional genotypes, culture media, and growth conditions in the aggregated dataset. It is also noteworthy that the publication describing our serial passage experiment reported a protein as significantly changing if its abundance in the ancestor strain was different from its average abundance in all the descendant lineages derived from those ancestors. For many proteins in that study, the abundances varied greatly among the descendant lineages. A significantly different average abundance between groups of cultures is therefore not necessarily the most useful metric for predicting group membership for individual cultures, as was the goal of this study.

When the LRC was trained with presence/absence data, it selected 12 proteins as features for the final model (S1 Table). Six of these proteins were also selected by the classifier when it used relative abundance data, and these six include three of the four that were identified as significantly changing in our previous study along with ATP synthase A chain. As in the classifier using relative abundance data, glucose-6-phosphate isomerase presence was strongly predictive of laboratory adaptation, while Ail and sulfite reductase were strongly predictive of wild strains.

### Testing the classifier

The LRC was very accurate in assigning wild or laboratory-adapted status to the samples using the replicated 10-fold cross-validation, in which 90% of the data was used for training and 10% for testing in each replicate. We therefore tried the more conservative approach of a two-fold cross-validation, using only 50% of the data to train the classifier and the remaining 50% to test it, repeated 100 times. As judged by two-fold cross-validation, the LRC built with presence/absence features had an estimated accuracy of 96.9% (standard deviation 2.2%); the LRC built with relative abundances had an estimated accuracy of 98.2% (standard deviation 2.1%). Thus when the classifier was trained with only half of the data, the accuracy in predicting the remaining half remained, on average, above 96%. This result demonstrates that the classifier is not unduly influenced by a small number of outlier datasets within the aggregated data.

**Fig 2:**
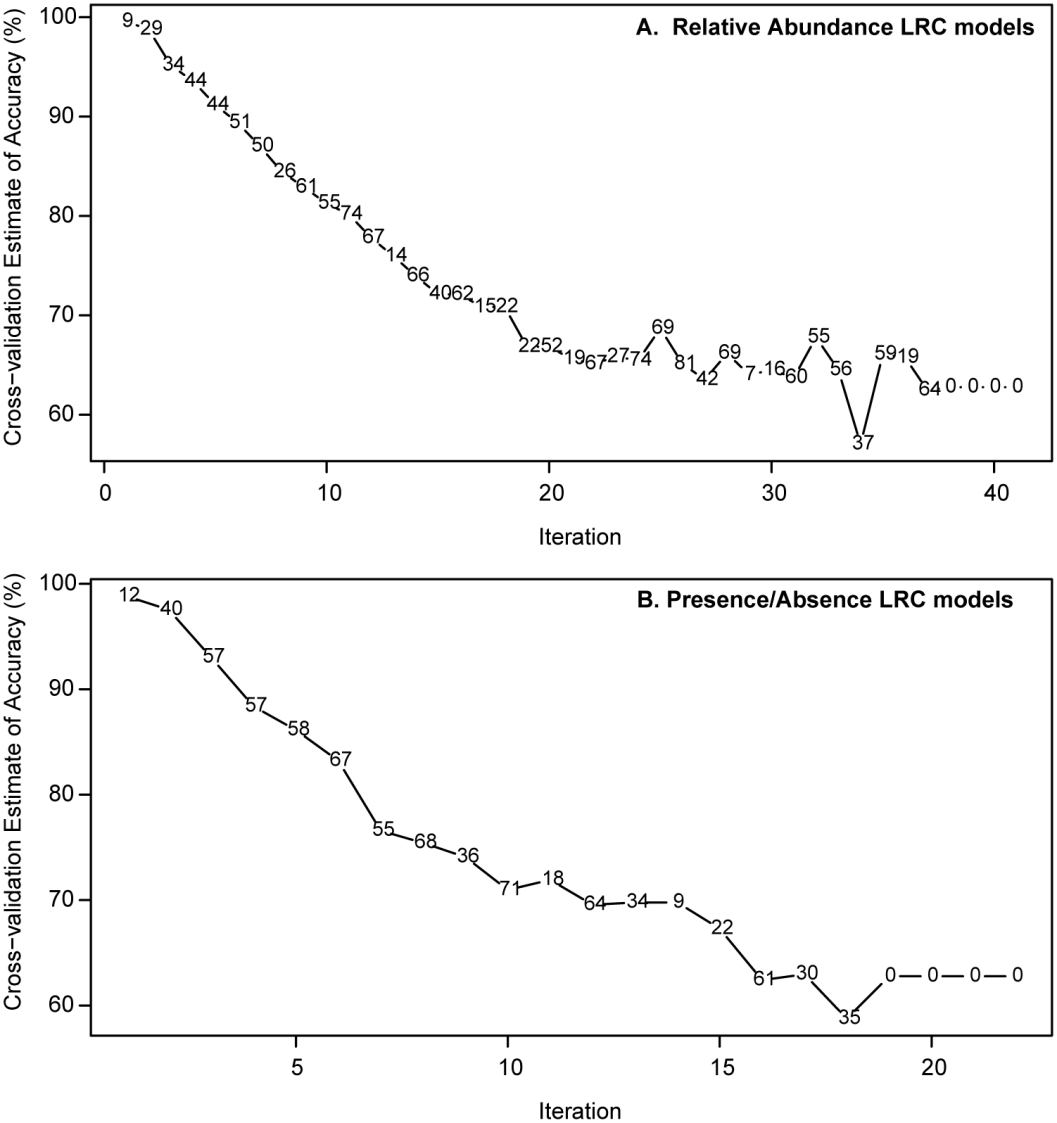
More protein features than those reported in Table 2 can accurately classify laboratory vs. wild samples. The Lasso logistic regression classifier (LRC) was constructed in iterations, with the input data for each iteration consisting of all protein features not selected by the LRC in any previous iteration. The plots show the classifier accuracy on the vertical axis plotted against the number of iterations on the horizontal axis. The number of features selected in each iteration is the plotted symbol. **A**, LRCs using quantitative protein abundance data; **B**, LRCs using presence/absence data. Note that the accuracy value in the limit of large numbers of iterations is equal to the proportion of laboratory samples in the data, and represents the limit where the features used contain no information useful for classification.

As a further test of the LRC, we iteratively retrained the classifier after removing the previous iteration’s selected features (proteins) from the input data. The results are shown in Fig 2. The classification accuracy remains above 90% for the first three iterations in the qualitative (discrete) model and the first five iterations in the quantitative model, suggesting that broad physiological changes occur during adaptation to laboratory conditions, and that abundance data of numerous proteins can serve to produce an effective classifier. Because our previous study found statistically significant changes in protein expression for 137 and 182 (union 249) proteins between the two wild isolates and their respective laboratory-adapted descendants, this result is unsurprising. Roughly 26% of the features selected in the first three iterations of the quantitative model were identified as significantly changing in the serial passaging experiment, as were ~35% of the qualitative features. As mentioned above, we do not expect full agreement between selected features in this study and differentially expressed proteins in our previous study because of the increased diversity of genotypes and growth conditions. However, some degree of overlap is an indication that there may be a common biological phenomenon underlying the signatures of domestication. In particular, glutamine synthetase and glutamate dehydrogenase (proteins with key functions in the metabolism of amino groups) appear both in the feature lists for the second and third iterations and in the serial passaging results, as does a NAD(P)H transhydrogenase subunit.

Significantly, after the first iteration, the number of selected features sharply increases, indicating that subsequent iterations require more proteins to achieve similar or lower levels of classification accuracy. Since the LRC is designed to select a small group of features that produces the most accurate classification, this observation is a sign that the method is working as expected. The presence of additional high-performing (although larger) feature sets highlights that there is rich information present in proteomics data.

**Fig 3:**
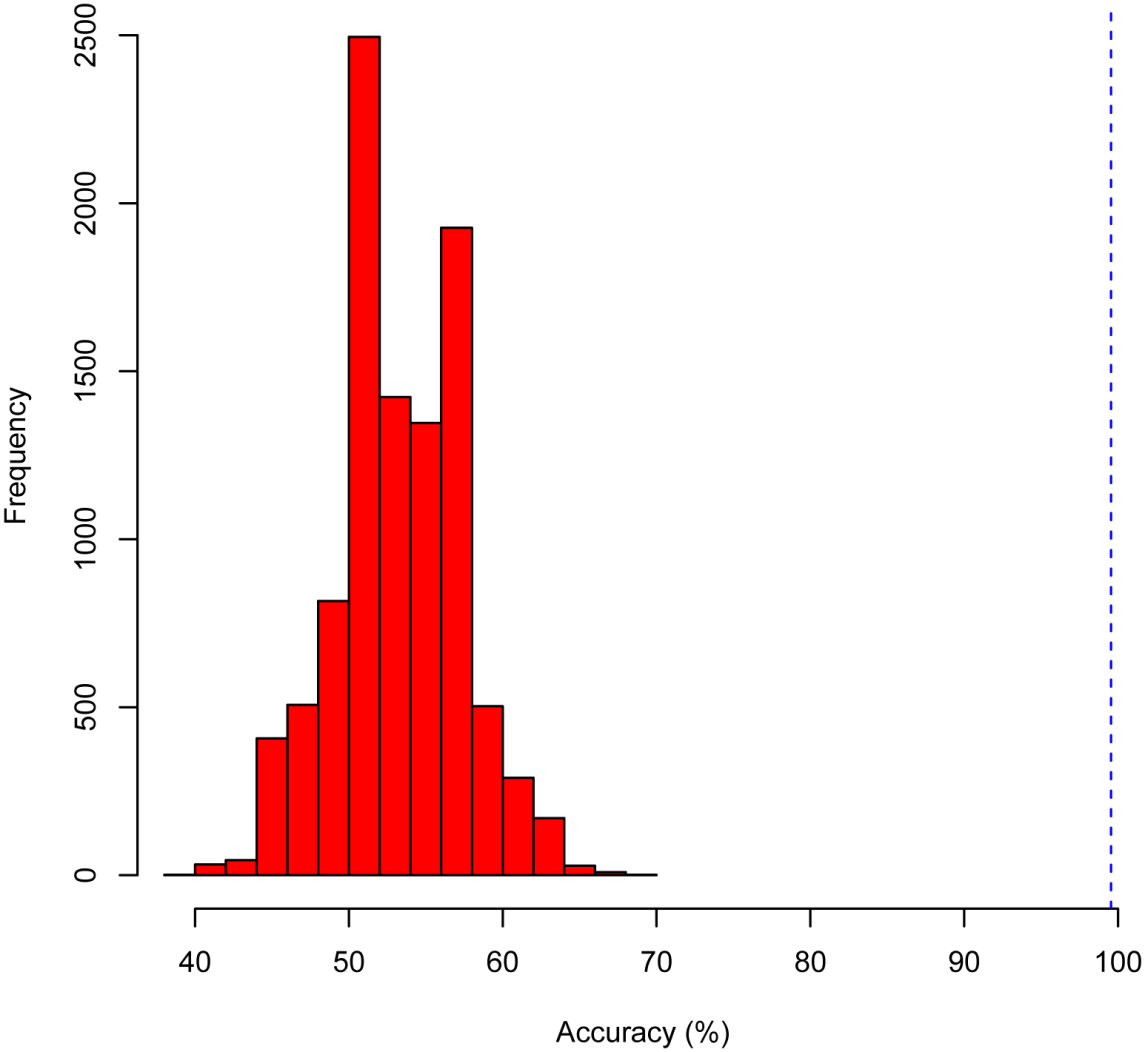
Illustration of the permutation test of the final LRC generated using 10-fold cross-validation and relative abundance features. The red histogram represents the distribution of the accuracy generated from 10,000 permutations of the laboratory-adapted/wild labels. This histogram represents the null, distribution, i.e., the distribution expected if no information relevant to distinguishing laboratory and wild samples were present in the data. The cross-validation estimate of the accuracy of the final LRC, 99.5% is illustrated by the dashed blue line. The distance of the blue line from the null distribution clearly indicates that the observed accuracy of the LRC did not occur by chance, supporting the conclusion that the data for laboratory-adapted and wild samples is truly different. Results for the other three LRC’s (2-fold with relative abundance, 10-fold with presence/absence, and 2-fold with presence/absence) were identical to this one.

As a final test of the LRC’s ability to distinguish wild from laboratory strains, we performed a permutation test [30] to determine whether the LRC had indeed identified a significant, or real, connection between the protein features and the strain (laboratory-adapted or wild)—as opposed to the relationship occurring by chance. We performed the permutation test for each of the four final LRC models (10-fold relative abundance, 2-fold relative abundance, 10-fold presence/absence, and 2-fold presence/absence). To perform the test, we created 10,000 permutations (i.e. randomizations) of the laboratory-adapted or wild labels in the data. We used a final LRC to predict the laboratory-adaptation status (using the appropriate features), and then calculated the accuracy of that prediction compared to each set of permuted strain labels. Hence, we obtained 10,000 estimates of the null distribution of the accuracy, i.e., the accuracy we would expect the LRC to produce if there were no relationship between laboratory/wild adaptation and the features. We then compared the cross-validation estimate of the accuracy of the final LRC (e.g. 99.5%, in the case of the LRC generated using 10-fold cross-validation and relative abundance features) to the corresponding null distribution of accuracy values. For all four LRC’s, the null distribution of the accuracy ranged between 30% and 62%, whereas the estimated accuracies for all four LRC’s was at least 96% (See Fig 3) This clearly demonstrates that the accuracy of the LRCs did not occur by chance, and we thereby conclude that there is a significant relationship between the expression of certain proteins and the laboratory-adapted or wild nature of the sample.

### Effect of genotype

The genotypes of the various strains in the samples presented potential confounding variables for the analysis. The samples belonged to two different biovars (subdivisions of the species *Y*. *pestis* based upon biochemical tests) and varied with respect to the presence of two recognized virulence factors, the CD1 plasmid and the 108 kb chromosomal pathogenicity island known as the pigmentation (*pgm*) locus [24]. *Y*. *pestis* strains have been historically categorized into 4 classical biovars, and DNA analysis has recently grouped them into major phylogenetic groups [31]. All of the wild isolates in this study, as well as the virulent laboratory strain CO92, belong to the molecular group 1.ORI (roughly corresponding to classical biovar orientalis), the only molecular group endemic to the U.S. The avirulent laboratory strain KIMD27 [14] is a member of the molecular group 2.MED and the classical biovar mediaevalis [31]. The wild *Y*. *pestis* isolates were recovered from prairie dog colonies that had died out from plague and were presumed to be fully virulent and therefore both *pgm*+ and CD1+, an assumption supported by whole genome sequencing of two of the isolates [12]. CO92, used in 41/86 of the laboratory-adapted samples, is also *pgm*+ and CD1+. KIMD27, used in 22/86 laboratory-adapted samples, has a deletion of the *pgm* locus, but retains CD1. Among the 23 laboratory-passaged populations generated in our serial passage study, fully 18 populations had lost the CD1 plasmid and 11 had deleted the *pgm* locus [12].

The classifiers generated by the Lasso suggested that neither biovar nor the presence of the two virulence factors was decisive for distinguishing wild versus laboratory-adapted strains. Of the nine proteins selected by the Lasso using relative abundance data (Table 2), only one (Biofilm PGA synthesis deacetylase PgaB, also known as HmsF) is encoded within the *pgm* locus, and none is encoded on CD1. None of the 12 proteins selected by the Lasso using presence/absence data was encoded within the *pgm* locus or on the CD1 plasmid. Finally, all of the KIMD27 samples (molecular group 2.MED) were classified correctly with the laboratory-adapted 1.ORI strains and separate from the wild 1.ORI strains by the LRC.

### Classification by analytical facility

The biomass samples represented in our data were inactivated by various methods, prepared in two different facilities, and measured by slightly different methods. Since all of the CO92 samples were prepared and run in Facility 2, and most of the wild strains were run in Facility 1, we were concerned that some of the “laboratory-adapted” signature might in fact be a signature of the analytical facility and all the associated differences in sample handling and data collection. (In fact, it was this concern that led us to reprocess and re-analyze several samples originally run in Facility 1 in Facility 2. Facility 2 data were used in the final classifier for these samples—see Table 1 and Fig 1B.) To address this concern, we trained the LRC again, this time to identify features that predict the laboratory facility. We first trained the LRC using the relative abundance data and again using the presence/absence data.

In both cases, the LRC was able to accurately (> 98% in 10-fold cross-validation) distinguish samples processed in Facility 1 from those processed in Facility 2. None of the four protein features selected by the LRC developed with relative abundance data (Table 3) overlapped those selected by the Lasso for classifying wild versus laboratory-adapted strains (Table 2). Of the 18 proteins selected as presence/absence features (S2 Table), only one overlapped with a feature selected to classify the wild versus laboratory-adapted state. This protein, ATP-dependent protease HslV was selected as one of the 12 presence/absence features (S1 Table), but not as a relative abundance feature (Table 2). Its coefficient in that classifier (S1 Table) is small; thus, its contribution to the prediction of wild versus laboratory-adapted is small relative to most of the other features. We therefore conclude that the analytical facility might have a small influence on the wild versus laboratory prediction when presence/absence data was used. However, in general, the proteomic signature for laboratory adaptation is different from the signature for the laboratory facility.

**Table 3:** Relative abundance protein features selected by the final logistic regression classifier to distinguish samples by analytical facility.

Protein name	Uniprot ID	Coefficient*
GMP reductase (EC 1.7.1.7)	Q8ZBI2	3.991
3-hydroxyacyl-[acyl-carrier-protein] dehydratase FabZ	Q8ZH57	2.4407
Putative exported protein	Q0WKJ6	1.2636
Superoxide dismutase [Cu-Zn] precursor (EC 1.15.1.1)	Q74XS8	0.8577

*The final LRC also includes an intercept term of -3.6159 and optimal tuning parameters of *λ* = 0.01643 and *τ* = 0.2. See Methods.

The fact that the proteomic data could be used to distinguish samples analyzed in the two different facilities is not altogether surprising. There were small but systematic differences in the sample preparation methods used by the two facilities: one of these was that samples analyzed in Facility 1 were inactivated by ethanol treatment (either before they were sent to the facility or in the facility itself), whereas inactivation of the samples analyzed in Facility 2 was mostly by treatment with 8M urea. We demonstrated previously that inactivating cells by ethanol treatment, autoclaving, or irradiation resulted in only minor changes in the detected proteome [20]. Urea treatment was not evaluated in that study, but the small number of proteins that could be used to distinguish samples analyzed in the two facilities is consistent with its general findings. Other potential differences could arise from the method of cell lysis (urea versus bead-beating), instrument settings (particularly data-dependent data acquisition settings), different chromatography gradients, and differences in instrumentation. The latter three of these differences would all tend to result in the acquisition of more tandem mass spectra, and consequently to the identification of more proteins, in the samples from Facility 2. Anecdotal evidence also suggests that there are variations over time and with sample preparation personnel, even within a single facility. Thus, the existence of a facility signature is not surprising.

### Proteomic signature of culture medium

We also tested whether the LRC could identify features that predicted the growth medium of a culture, and if so, whether the signature for culture medium was similar to the signature for wild versus laboratory-adapted strain. The composition of the growth medium is known to affect gene expression and thus protein abundances, and was potentially another confounding factor in our analysis. In our aggregated datasets, the BCS and DMEM media were used only with laboratory strains, while BHI and TSB were used for multiple cultures of both wild and laboratory-adapted strains (Table 1, Fig 1C). We used an approach similar to testing the signature for the laboratory facility: we trained the classifier [in this case, a Lasso multinomial logistic regression model [7]] to predict the culture medium, once using the relative abundance data, and again using the presence/absence data. The classifier developed with relative abundance data selected 29 protein features to predict culture medium (S3 Table), while the classifier developed with presence/absence data selected 35 features (S4 Table). These two classifiers had 12 protein features in common, only one of which (hypothetical flavoprotein Q7CH13) was also selected by the Lasso LRC to predict wild versus laboratory-adapted samples in the presence/absence model (S1 Table). Thus out of 52 total protein features selected to predict culture medium between the two models, only one was also selected in one model to predict wild vs. laboratory-adapted strain. The relative lack of overlap in protein features that distinguish growth medium versus laboratory adaptation suggests that the general biological response of *Y*. *pestis* to laboratory adaptation is distinct from its response to its growth medium.

In general, the relatively large number of protein features selected to classify culture medium included proteins involved in carbohydrate and amino acid metabolism, iron acquisition, fatty acid and vitamin biosynthesis, and transport. The protein features selected by the classifier developed with presence/absence data also included some amino acid metabolism enzymes, but included more transporters and several proteins that function in redox maintenance and oxidative damage repair. It is not surprising that expression of these types of proteins would change in response to culture medium. The expression of many genes in *Y*. *pestis*, including virulence factors, has been shown to respond to concentrations of calcium and iron [27, 32], and the various complex media used to produce the samples represented in our aggregated dataset undoubtedly varied in concentrations of these two elements. The proteins selected by the LRCs to classify culture medium also included proteins of the galactose utilization pathway, suggesting that the media varied significantly in the concentration of this sugar.

Interestingly, three proteins involved in amino acid metabolism noted to have changed significantly during our serial passage experiment appeared in the list of features selected by the growth medium classifier that was developed with presence/absence features: anthranilate synthase aminase component, urease gamma subunit, and a putative periplasmic solute-binding protein (see Leiser et al, Table 2). In the serial passage experiment, which was conducted in BHI, anthranilate synthase was detected only in the ancestor populations of one of the two strains, while the other two proteins increased in abundance with serial passaging [12]. Similarly, the growth medium classifier showed anthranilate synthase having a negative coefficient for growth in BHI, while the coefficients of the other two proteins were positive, in particular the coefficient for urease gamma subunit. Neither of the LRCs developed to classify wild versus laboratory adapted strains identified any of these three proteins as a classifying feature. This result suggests that the changes in expression of these three proteins observed during our earlier serial passage experiment were likely an effect of prolonged growth in BHI and not a general response to laboratory adaptation.

## Discussion

We have shown that a machine learning tool, the Lasso logistic regression classifier, can successfully distinguish biomass from minimally-cultured wild strains of *Y*. *pestis* and biomass of long-term laboratory strains using mass spectrometric proteomic data. The classifier was highly accurate when using either presence/absence data or relative abundance measurements. Further, additional, largely non-overlapping feature sets related to other characteristics of the samples could also be extracted. Previous proteomics-related applications of the Lasso methodology include inference of proteins present in a sample from detected peptides [33] and data set quality control in a high-throughput environment [34]. In another recent study, Dammeier et al. [35] applied different machine learning tools to develop proteomic signatures of various tissues from residual material on the surfaces of bullets. Those investigators also used both presence/absence data and relative protein abundance data to develop highly accurate classifiers. A key difference between their work and ours is that their classifiers were derived from a designed set of controlled experiments, whereas we used archived data from diverse experiments that were not designed to address the characteristics we tested.

Our results suggest that protein features that distinguish wild from laboratory strains of *Y*. *pestis* transcend the experimenter group, organism genotype, growth medium, growth temperature, time of culture, details of sample preparation, and the instrument and facility of analysis. More broadly, our work suggests it will be possible to mine public proteomic data repositories to extract information unrelated to the original intent of the experiments, and thus extend the utility of such data.

### Adaptation to laboratory growth

It is important to emphasize that all of the cultures used in our study were grown in laboratory media, and that the identity of the medium was not important in the classification of wild vs. laboratory-adapted strains. Therefore, the changes in protein abundance or presence/absence selected by the classifier are unlikely to be immediate regulatory responses to the composition of the culture medium. We made a similar observation during our serial passage experiment, where we observed significant changes in protein abundances with serial passaging, even though the ancestor and descendant strains were all grown in the same laboratory medium. Rather, our results suggest that *Y*. *pestis* adapts to long-term laboratory culture by altering expression levels of a suite of proteins by a mechanism or mechanisms beyond simple gene regulation.

The classifier functions to select a minimal number of features with maximum predictive power, so features that contain redundant information are not likely to be selected. The analysis in Fig 2 shows this clearly. Therefore, unlike traditional proteomics expression level analysis, the LRC methodology is not likely to highlight whole pathways or processes that are coordinately regulated. A clear biological model would provide confidence in the results, insights that lead to improved measurements, better understanding of limitations, and potentially transferability to other organisms. Unfortunately, studies of the fundamental biology of *Y*. *pestis* are lacking, making interpretation of our data difficult. For example, nitrogen metabolism in *Y*. *pestis* differs from that of model enteric bacteria because of both different regulatory circuitry (*Y*. *pestis* lacks the *nac* transcription factor present in *E*. *coli* and *Klebsiella pneumonia* [29, 36–38]) and multiple mutations in the *Y*. *pestis* genome [39–42] and remains uncharacterized In the absence of a clear molecular basis for interpretation, we will limit discussion of biological mechanism to the following general observations.

Many of the proteins in Table 2 whose relative abundance levels are positively correlated with the probability of laboratory adaptation (i.e., proteins with positive coefficients) are involved in central carbon and energy metabolism, and one (chorismate mutase) is an enzyme involved in amino acid synthesis The abundances of proteins involved in central carbon metabolism and amino acid metabolism also changed significantly during serial passaging of wild isolates of commensal enterobacteria in rich laboratory media [43], as in our serial passaging experiment with *Y*. *pestis* [12]. These changes can be rationalized as a means for the organism to better use the abundant and varied nutritional resources in the rich laboratory media in which they were grown.

It is interesting to note that two of the nine features listed in Table 2 are redox proteins: the *E*. *coli* DsbA protein carries out redox-dependent oxidative folding of disulfide-bonded proteins [44], while the NADPH-sulfite reductase of *E*. *coli* can reduce sulfite and other substrates [45]. It is possible that the changes in abundances of redox proteins are associated with the general change in metabolism just described. One might also speculate that rich medium leads to a higher rate of oxidative metabolism and associated oxidative stress, requiring changes in cellular responses.

Increased levels of the virulence factor attachment invasion protein (Ail) were associated with increase probability of a sample being wild. Decreased Ail expression and genomic mutations that led to truncated Ail protein were observed in our serial passaging experiment. Ail is a very highly expressed protein in *Y*. *pestis* [46, 47]. It therefore logical that in laboratory conditions, where Ail confers no survival advantage, cells can increase fitness by decreasing Ail expression and thus reducing metabolic load. A similar argument could be made for other virulence factors, but reduced Ail expression might have a more dramatic effect on fitness in culture because Ail is so abundant. PgaB/HmsF, which is essential for biofilm production in the flea, might fall into this category as well, although genotype (HmsF is part of the *pgm* locus) and culture temperature are possible confounding factors for expression of this gene as well [48].

Many of the protein abundance changes observed in our earlier study of *E*. *coli* adaptation could be attributed to the accumulation of mutations in the global regulatory genes *arcA* and *rpoS* [43]. In contrast, most of the protein abundance changes we observed during our serial passage experiment with *Y*. *pestis* could not obviously be associated with genome changes. Reasons that the associations were not obvious could include lack of understanding of regulatory circuitry in *Y*. *pestis*, mutations in intergenic regions with as yet unrecognized consequences, or epigenetic changes. Indeed, the discovery of protein abundance features that distinguish laboratory-adapted and wild strains of *Y*. *pestis* can provide a basis for hypothesis-driven research into the mechanisms behind the changes, as well as the biological roles of the adaptations. Regardless of the mechanism, the evidence from these two previous studies and our current result suggests that changes in the abundance of proteins related to resource usage may be a hallmark of adaptation to prolonged growth in rich laboratory media.

### Distinguishing laboratory-grown and naturally-occurring pathogens

Differentiating naturally-occurring pathogens from laboratory-adapted strains of the organism has remained a challenge for the biodefense community. All of the bacteria designated as Select Agents by the U.S. Centers for Disease Control and Prevention are naturally-occurring organisms. The implications of detecting such a pathogen could be quite different if it is a wild versus a laboratory strain, and its genome sequence may or may not be useful in making this distinction. For example, two tourists in New York City became ill with plague in November, 2002 [49]. As plague does not occur naturally in the eastern United States, and these individuals became ill in a major US city, these infections raised concerns about a potential bioterrorism attack. However, investigators were ultimately able to attribute the infections to organisms acquired near the individuals’ home in northern New Mexico by comparing multi-locus DNA genotypes of a clinical isolate from one of the patients to environmental *Y*. *pestis* isolated from fleas near their home [50]. The analysis was very persuasive, but the approach was lengthy and would rarely be practical, as it would not always be possible to obtain the type of environmental samples that were used in this study to compare to the patient isolates [51]. And even if near genetic relatives could be identified among wild isolates, genome sequence data alone may not reveal whether the organism in question had been isolated in that area and then used as a laboratory strain. Our results suggest that protein abundance measurements might provide a direct, measurable signature of laboratory adaptation and could lead to faster, more confident determinations without requiring the availability of matched environmental samples.

The use of protein abundances to distinguish wild from laboratory strains requires additional testing. This initial effort was essentially opportunistic: we used all the relevant data to which we had access. This led to some weakesses in our dataset. For instance, we had only two standard laboratory strains: KIMD27 and CO92. A total of 23 of the 95 cultures of laboratory-adapted strains were descendants of two wild isolates that had undergone 60 serial transfers in the laboratory. These strains are arguably not representative of standard laboratory strains, even though the classifier was used successfully with multiple samples of the standard virulent CO92 (41 independent cultures under varying conditions) and the standard avirulent KIMD27 (19 independent cultures under varying conditions). Tests with numerous additional standard laboratory strains will be very important to confirm the general utility of the approach.

Validation would entail acquiring proteomics data on a large number of independent wild *Yersinia pestis* samples including new wild isolates (cultured in controlled conditions with no confounding variables), and constructing a classifier with only some of the samples, reserving some for a true external validation. Targeted assays (mass spectrometric or immunological) could then be developed for proteins selected by the classifier, and measurements conducted on an even larger, blinded set of new wild and laboratory-adapted samples, and the statistical performance evaluated.

Ideally, other studies would, in parallel, illuminate the underlying mechanisms by which organisms adapt to laboratory conditions The process of “domestication” of a wild pathogen involves genetically programmed regulatory responses, and evolutionary changes, probably including evolution of the regulatory networks themselves. The heterogeneity observed in our serial passaging experiments is consistent with convergent evolution towards an adaptive phenotype. Characterizing this common adaptive phenotype and pathways leading to it is critical for increasing confidence of classification results. A mechanistic focus would also have the advantage of providing insights applicable to multiple organisms.

### Mining archived proteomic data

The ability to mine mixed, archived datasets for multiple signatures would be a boon to biomarker discovery efforts in many contexts. Despite all of the above-noted variables in sample production, preparation, and analysis, all of the data could be described as having been collected by standard bottom-up (i.e., peptide-based) proteomic methods. We were able to re-analyze the mass spectrometric data and extract potentially useful information. The scientific utility of public DNA sequence databases is well recognized; our work suggests that properly curated proteomic databases that link datasets to metadata may be similarly useful. Furthermore, while it is still not possible to perform accurate quantitative comparisons on disparate datasets [9, 10], the logistic regression classifier technique used here illustrates how quantitative information from archived data can be leveraged for signature and biomarker discovery. For instance, one could envision retrospectively mining datasets of human plasma proteome studies for biomarkers for a disease other than those targeted by the original studies. Associating comprehensive metadata with the mass spectrometric data will be essential to support such analyses. Targeted follow-up studies could then test the hypotheses generated by data mining.

### Conclusions

The work presented here should be viewed as preliminary proof-of-concept for a novel use of proteomics data, particularly archived and repurposed proteomics data, and a novel application of statistical learning methodology in proteomics. Our major findings are (1) that the process of adaptation to laboratory conditions, although still not well-characterized, is more general than the narrow case of our previous serial passaging studies, (2) that proteomics datasets, even those acquired for unrelated studies, contain the information needed to classify samples as laboratory-adapted or wild. Our work suggests that proteomics datasets could be mined for purposes independent from those of the original experiments, and that public reservoirs of proteomic data, if accompanied by sufficiently detailed metadata, could be rich resources for future scientific discovery Although we have attempted to carefully evaluate our results, particularly in light of the known confounding variables, our evaluation has focused on the output of the classifier, and not on the selected features. We have not attempted a full formal validation of the candidate proteins as biomarkers as is commonly done for proteomics or other candidate biomarkers of human disease.

## Supporting information

S1 Dataset Detailed sample metadata. (XLSX)

S1 Fig Output of the LRC to distinguish wild from laboratory-adapted strains using presence/absence data. Each symbol represents the prediction of the LRC for an independent culture. Triangles represent cultures of wild strains. Circles represent laboratory-adapted strains. The horizontal axis value is the predicted probability that a culture is laboratory adapted and is non-linear; points are separated vertically in a random fashion to improve the visualization. See Methods for an explanation of τ. A. Colors represent wild versus laboratory-adapted. B. Colors represent the facility of preparation and analysis. C. Colors represent the laboratory medium in which the cultures were grown prior to analysis.
(TIF)

S1 Methods Data preparation [19]. (DOCX)

S2 Methods Lasso regression classifier [7, 52–54]. (DOCX)

S1 Table Protein features that predict wild versus laboratory strains selected by the LRC using presence/absence data. (DOCX)

S2 Table Protein features that predict analytical facility selected by the LRC using presence/absence data. (DOCX)

S3 Table Protein selected by the Lasso to classify samples by growth medium using relative abundance data. (DOCX)

S4 Table Proteins selected by the Lasso to classify samples by growth medium using presence/absence data. (DOCX)
