# Supplementary figures and images for: Protein abundances can distinguish between naturally-occurring and laboratory strains of Yersinia pestis, the causative agent of plague
Source: PLoS One. 2017 Aug 30;12(8):e0183478. doi: 10.1371/journal.pone.0183478 (PMC5576697; doi:10.1371/journal.pone.0183478)

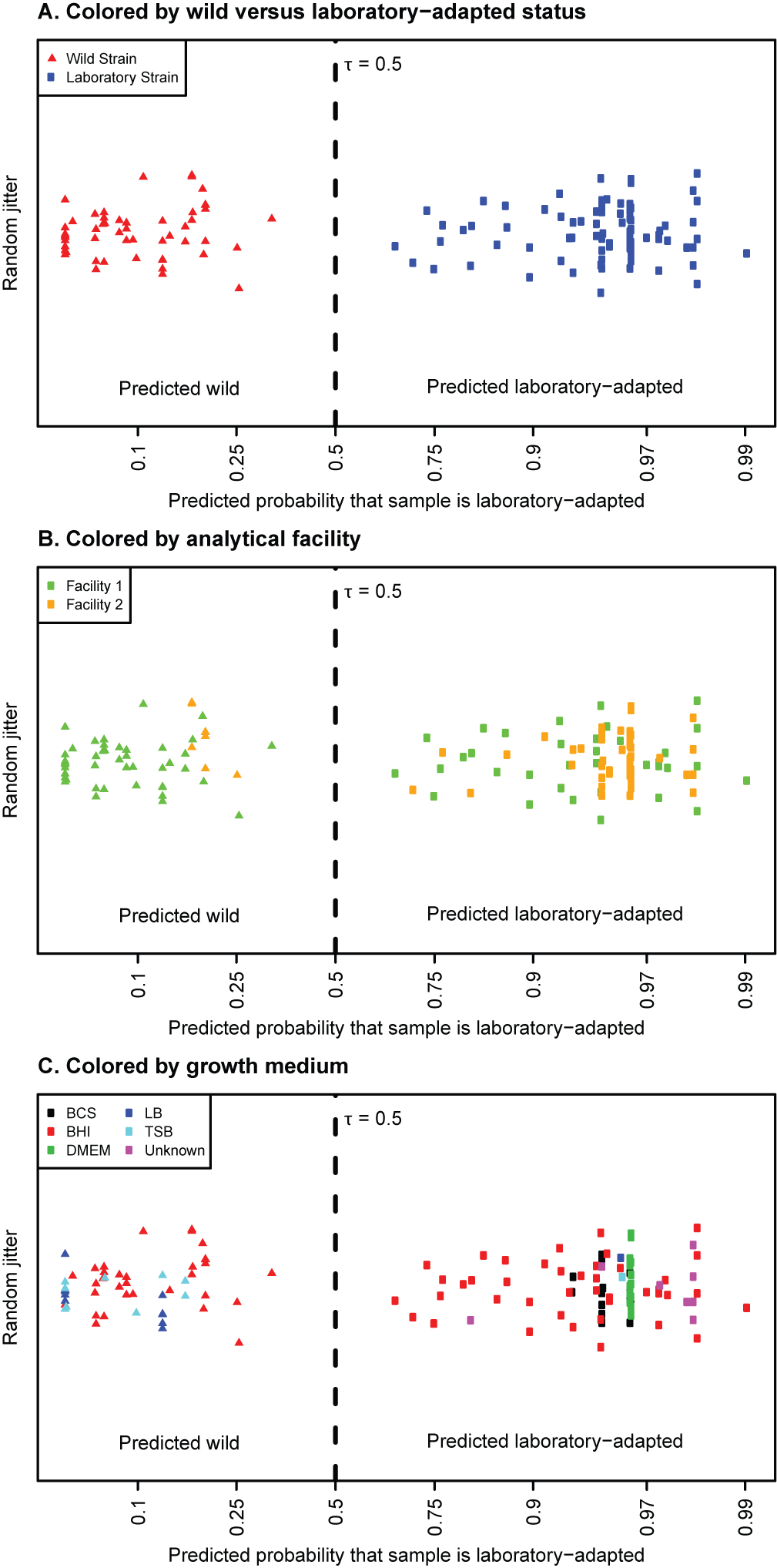

Supplement: S1 Fig — Each symbol represents the prediction of the LRC for an independent culture. Triangles represent cultures of wild strains. Circles represent laboratory-adapted strains. The horizontal axis value is the predicted probability that a culture is laboratory adapted and is non-linear; points are separated vertically in a random fashion to improve the visualization. See Methods for an explanation of τ. A. Colors represent wild versus laboratory-adapted. B. Colors represent the facility of preparation and analysis. C. Colors represent the laboratory medium in which the cultures were grown prior to analysis. (TIF) [file pone.0183478.s002.tif]
