# Supplementary material for: Protein abundances can distinguish between naturally-occurring and laboratory strains of Yersinia pestis, the causative agent of plague
Source: PLoS One. 2017 Aug 30;12(8):e0183478. doi: 10.1371/journal.pone.0183478 (PMC5576697; doi:10.1371/journal.pone.0183478)
Supplement: S1 Methods — (DOCX) [file pone.0183478.s003.docx]

**Methods: Data preparation**

*Y. pestis d*atasets generated for this endeavor and archived *Y. pestis* datasets from two administratively distinct and physically separated research groups at PNNL were pooled together. The datasets were derived from *Y. pestis* protein samples that had been digested with trypsin; we did not use data from samples that were fractionated prior to mass spectrometric analysis. The resulting set of 381 datasets originated from 137 samples produced for a wide range of experiments conducted over the course of several years. The full set of datasets was split into six different groups for practical reasons (i.e., computational run time and memory limits, and differences in sequence database and modifications); each group was analyzed by a separate MaxQuant session [([40](#_ENREF_40)) version 1.5.1.2] to identify and quantify proteins present in each individual dataset. MaxQuant parameters were set to their default values, except re-quantify was set to true. Datasets in MaxQuant sessions 1 and 2 contained data from the KIMD27 strain and were searched against a *Y. pestis* KIM10+ database. Datasets in MaxQuant sessions 3, 4, 5, and 6 contained data from CO92, the wild isolates, and the serially-passaged strains and were searched against a *Y. pestis* CO92 database. All sessions were run with the following variable modifications: methionine oxidation and acetylation of the protein amino terminus. Sessions 2, 3, 4, and 5 were run with a fixed carbamidomethyl modification. Protein identifiers from the KIM10+ database and the CO92 database were mapped to each other using BLAST. Custom scripts were used to collect and combine the protein abundances of all proteins from all sessions into a single file consisting of a matrix where each row corresponded to a protein identifier, each column corresponded to a dataset, and each cell contained the intensity of a particular protein in a particular dataset. Missing values (non-detected proteins) were set to 0. This matrix was used to derive the presence/absence features and the quantitative features (i.e., the transformed abundance scores) that are described in detail in Results. Metadata from each of the 381 datasets is presented in Dataset 1 of the Supporting Information.
