# Supplementary material for: Protein abundances can distinguish between naturally-occurring and laboratory strains of Yersinia pestis, the causative agent of plague
Source: PLoS One. 2017 Aug 30;12(8):e0183478. doi: 10.1371/journal.pone.0183478 (PMC5576697; doi:10.1371/journal.pone.0183478)
Supplement: S2 Methods — (DOCX) [file pone.0183478.s004.docx]

**Methods: The Lasso Regression Classifier**

We present in detail the construction of the Logistic Regression Classifier (LRC). The calculations were performed using the *glmnetLRC [1]* package in R [2]. We begin by defining the index variables. Let $i=1,\ldots,N$ index the number of biological samples, or cultures. Let $j=1,\ldots,2068$ index the proteins (i.e., features) used in the LRC. Let $k=1,\ldots,n_{i}$, index the technical replicates of sample $i,$ where the value of $n_{i}$ is typically 3. Let $y_{i}=0$ indicate that biological sample $i$ is a wild strain*,* and $y_{i}=1$ indicate the sample is laboratory strain. Each sample also contains a feature vector with 2,068 elements denoted by $\boldsymbol{x}_{i}=\left( x_{i,1}, x_{i,2},\ldots,x_{i,2068} \right)^{T}$.

For the quantitative LRC based whose features were transformed relative abundance scores, we used the following formulae, where the quantitative features, $x_{ij},$ are derived from the raw abundance measures, $w_{ijk}:$

$$z_{ijk}=\log_{2} \left( w_{ijk}+1 \right), z_{i}^{'}=\min_{\begin{aligned} 1\leq k\leq n_{i} \\ 1\leq j\leq2068 \end{aligned}} z_{ijk}, z_{i}^{''}=\max_{\begin{aligned} 1\leq k\leq n_{i} \\ 1\leq j\leq2068 \end{aligned}} z_{ijk}$$

$$x_{ij}=\frac{1}{n_{i}}\sum_{k=1}^{n_{i}} \left( \frac{z_{ijk}-z_{i}^{'}}{z_{i}^{''}-z_{i}^{'}} \right)$$

For the LRC whose features were presence/absence, we used the following formulae to calculate the features, $x_{ij}:$

$$u_{ijk}=\left\{ \begin{matrix} 1 if protein j is present in sample i and technical replicate k \\ 0 if protein is absent \end{matrix} \right.$$

$$x_{ij}=\left\{ \begin{matrix} 1 \mathrm{if} \sum_{k=1}^{n_{i}} u_{ijk}>0 \\ 0 \mathrm{otherwise} \end{matrix} \right.$$

The logistic regression model is given by:

$$P\left( y_{i}=1 \right):=\pi_{i}=\frac{\exp\left( \beta_{0}+\boldsymbol{\beta}_{1}^{T}\boldsymbol{x}_{i} \right)}{1+\exp\left( \beta_{0}+\boldsymbol{\beta}_{1}^{T}\boldsymbol{x}_{i} \right)}$$

where $\beta_{0}$ is an intercept term, $\boldsymbol{\beta}_{1}\boldsymbol{=}\left( \beta_{1}\boldsymbol{,\ldots,}\beta_{2068} \right)^{T}$ is a vector of logistic regression coefficients, and for notational convenience, , $\boldsymbol{\beta=}\left( {\beta_{0},\beta}_{1}\boldsymbol{,\ldots,}\beta_{2068} \right)^{T}$**.** The estimate of the vector of regression parameters, $\boldsymbol{\beta}$, is influenced by a tuning parameter $\lambda$. For this reason, we will often write $\boldsymbol{\beta}$ as $\boldsymbol{\beta(}\lambda\boldsymbol{)}$**.** The value of $\lambda>0$ controls the weight of the penalty of the log-likelihood function. An additional tuning parameter, $\tau\in\left( 0,1 \right),$ provides a threshold for the LRC such that if $\pi_{i}>\tau,$biological sample $i$ is predicted to belong to the laboratory strain.

When we fit the lasso logistic regression model to the data, we obtain $\hat{\boldsymbol{\beta}}\boldsymbol{(}\lambda\boldsymbol{)}$, and estimate of $\boldsymbol{\beta(}\lambda\boldsymbol{)}$. Therefore, let $\hat{\pi}_{i}:=\pi(\boldsymbol{x}_{i},\hat{\boldsymbol{\beta}}\boldsymbol{(}\lambda\boldsymbol{)} )$ denote the predicted probability that $y_{i}=1$ (i.e., that sample $i$ comes from the laboratory strain). Then, if $\hat{\pi}_{i}>\tau$, the LRC predicts that $y_{i}=1$, otherwise it predicts $y_{i}=0.$ For notational convenience, let $I(A)$ denote the indicator function, such that $I\left( A \right)=1$ if $A$ is true and $I\left( A \right)=0$ if $A$ is false. It will be useful to represent the predicted class of sample $i$ as $\hat{y}_{i}=f\left( \boldsymbol{x}_{i},\hat{\boldsymbol{\beta}}\left( \lambda\right)\boldsymbol{,}\tau\right)=I(\hat{\pi}_{i}>\tau)$, where $f$ can be thought of as the LRC. For the lasso, the estimate $\hat{\boldsymbol{\beta}}(\lambda)$ is the $\boldsymbol{\beta}$ that maximizes the penalized, binomial log-likelihood function:

$\hat{\boldsymbol{\beta}}\left( \lambda\right)\boldsymbol{=}\arg\max_{\boldsymbol{\beta}\in\mathbb{R}^{2068}} \left[ \frac{1}{N}\sum_{i=1}^{N} \left\{ y_{i}\left( \beta_{0}+\boldsymbol{\beta}_{1}^{T}\boldsymbol{x}_{i} \right)-\log(1+\exp(\beta_{0}+\boldsymbol{\beta}_{1}^{T}\boldsymbol{x}_{i})) \right\}-\lambda\sum_{j=1}^{2068} \left| \beta_{j} \right| \right]$ [1]

The optimal values of the tuning parameters, $\lambda$ and $\tau$, are obtained by minimizing the risk, or expected loss, of the LRC, where the risk is calculated via cross validation. Calculating risk requires that we define a loss function, $L(y,\hat{y})$. In this case, we employed the commonly used 0-1 loss, where the loss is 0 if the LRC predicts correctly, and 1 the LRC predicts incorrectly. We can write this as $L\left( y,\hat{y} \right)=I(y\neq\hat{y})$.

Cross validation is accomplished by randomly partitioning the data into $M$ folds (non-overlapping and exhaustive subsets), where each fold is tested using a model trained on the remaining folds. Following the notation of [3], let

$\delta:\left\{ 1,\ldots,N \right\}\to\{1,\ldots,M\}$ [2]

map each observation in the data to one of the folds. Let ${\hat{\boldsymbol{\beta}}}^{-m}\boldsymbol{(}\lambda\boldsymbol{)}$ represent the estimate of $\boldsymbol{\beta}$ obtained by fitting the lasso logistic regression model to all the data except the $m$^th^ fold. The cross validation estimate of the risk is given by

$R\left( \lambda,\tau\right)=\frac{1}{N}\sum_{i=1}^{N} I\left( y_{i}\neq f(\boldsymbol{x}_{\boldsymbol{i}}, {\hat{\boldsymbol{\beta}}}^{-\delta\left( i \right)}\left( \lambda\right)\boldsymbol{,}\tau) \right)$ [3]

The optimal estimates of the tuning parameters are those that minimize the risk:

$$\left( \hat{\lambda},\hat{\tau} \right)=\arg\min_{\lambda,\tau} R(\lambda, \tau)$$

In practice, we calculate $\left( \hat{\lambda},\hat{\tau} \right)$ by computing $R\left( \lambda,\tau\right)$ over a ragged matrix of discrete parameter values, defined by the combination of two vectors: $\boldsymbol{\lambda}\times\boldsymbol{\tau}$. The point in the matrix that minimizes the risk becomes the estimate for $\left( \lambda, \tau\right).$ In the event there are ties for the lowest risk for two or more points in the matrix, points with $\tau$ nearer to 0.5 are preferred, and if that still does not break the tie, points with larger values of $\lambda$are preferred because they result in a more parsimonious model with fewer predictors. In our case, we set $\boldsymbol{\tau}={(0.05, 0.10, \ldots, 0.90)}^{T}$ and the value of $\boldsymbol{\lambda}$ was chosen algorithmically by the default values in the *glmnet* package [4].

So far in this discussion, we have made reference to a single random partition, $\delta,$ of the data into $M$ folds. Naturally, the estimates of the tuning parameters depend on the partition. A different partition will yield different estimates of the tuning parameters. To ensure the final LRC is robust to the random partitioning process, we repeated the training process for 100 different random partitions (which we write as $\delta_{1},\ldots,\delta_{100}$) producing estimates $\left( \hat{\lambda}_{\mathcal{l}},\hat{\tau}_{\mathcal{l}} \right)$ for $\mathcal{l=}1,..,100.$ We refer to the repetition of the cross validation estimates as *cross validation replication*.

Once $\hat{\lambda}$ and $\hat{\tau}$ are identified for all the cross validation replicates, the final estimate of the tuning parameters is obtained by calculating the median of each one separately:

$\left( \hat{\lambda}^{*}, \hat{\tau}^{*} \right)=(\underset{\mathcal{l}}{\mathrm{median}} \hat{\lambda}_{\mathcal{l}}, \underset{\mathcal{l}}{\mathrm{median}} \hat{\tau}_{\mathcal{l}})$ [4]

The final estimator, $\hat{\boldsymbol{\beta}}\left( \hat{\lambda}^{*} \right)\boldsymbol{,}$ is obtained by fitting all the training data (via equation [1]) using the final estimates of the tuning parameters in equation [4], which gives rise to the final LRC:

$$f^{*}:=f(\boldsymbol{x}_{i},\hat{\boldsymbol{\beta}}\left( \hat{\lambda}^{*} \right)\boldsymbol{,}\hat{\tau}^{*} )$$

The coefficients shown in Tables 2, 3, S2, and S3 are from $f^{*}$, i.e., they are the values of $\hat{\boldsymbol{\beta}}\left( \hat{\lambda}^{*} \right)$**.**

The overall estimate of the accuracy of $f^{*}$ derives from the cross-validation estimates of the risk, averaged over the cross validation replicates. Using the final tuning parameter estimates in equation [4], a corresponding set of logistic regression parameter estimates ${\hat{\boldsymbol{\beta}}}_{\mathcal{l}}^{-m}\boldsymbol{(}\hat{\lambda}^{*}\boldsymbol{)}$ were obtained using equation [1] for each of the $M$ folds in cross validation replicate $\mathcal{l}$. The estimate for the risk for cross validation replicate $\mathcal{l}$ is given by applying equation [3] as follows:

$R_{\mathcal{l}}=\frac{1}{N}\sum_{i=1}^{N} I\left( y_{i}\neq f(\boldsymbol{x}_{\boldsymbol{i}}, {\hat{\boldsymbol{\beta}}}_{\mathcal{l}}^{-\delta_{\mathcal{l}}\left( i \right)}\left( \lambda\right)\boldsymbol{,}\tau) \right)$

The value of $R_{\mathcal{l}}$ is calculated for $\mathcal{l=}1,\ldots,100$ and summarized using the mean and standard deviation in the usual way:

$\bar{R}=\frac{1}{100}\sum_{\mathcal{l=}1}^{100} R_{\mathcal{l}}, \sigma_{R}=\sqrt{{\sum_{\mathcal{l=}1}^{100} \left( R_{\mathcal{l}}-\bar{R} \right)^{2}}/{99}}$

For easier interpretability, we report the cross validation estimate of the risk, $\bar{R}$, as the overall estimated accuracy, $1-\bar{R}$, which also has a standard deviation of $\sigma_{R}$.

1. Sego L, Venzin A, JA R. glmnetLRC: Lasso and Elastic-Net Logistic Regression Classification (LRC) with an Arbitrary Loss Function in R. Pacific Northwest National Laboratory2015.

2. R Core Team. R: A Language and Environment for Statistical Computing. Vienna, Austria2015.

3. Hastie T, Tibshirani R, Friedman J. The Elements of Statistical Learning: Data Mining, Inference, and Prediction, 2nd Edition. New York, NY: Springer-Verlag; 2009. p. 119-27.

4. Friedman J, Hastie T, Tibshirani R. Regularization Paths for Generalized Linear Models via Coordinate Descent. Journal of Statistical Software. 2010;33(1):1-22.
