# Supplementary material for: Protein abundances can distinguish between naturally-occurring and laboratory strains of Yersinia pestis, the causative agent of plague
Source: PLoS One. 2017 Aug 30;12(8):e0183478. doi: 10.1371/journal.pone.0183478 (PMC5576697; doi:10.1371/journal.pone.0183478)
Supplement: S1 Table — (DOCX) [file pone.0183478.s005.docx]

**Table S1. Protein features selected to distinguish wild and laboratory-adapted *Y. pestis* in the Logistic Regression Classifier using presence/absence protein data**

| **Protein** | **Uniprot accession** | **LRC Coefficient** |
| --- | --- | --- |
| Periplasmic chorismate mutase I precursor (EC 5.4.99.5) | Q7CHH5 | 2.198 |
| Glucose-6-phosphate isomerase (EC 5.3.1.9) | Q8ZAS2 | 1.3464 |
| Periplasmic thiol:disulfide interchange protein DsbA | Q9XBV2 | 0.4467 |
| ATP synthase A chain (EC 3.6.3.14) ^ϮϮ^ | Q7CFM3 | 0.3361 |
| C-terminal domain of CinA type S; Protein Implicated in DNA repair function with RecA and MutS; CinA is the first gene in the competence-inducible operon | Q0WBY4 | 0.3174 |
| Hypothetical flavoprotein | Q7CH13 | 0.1511 |
| ATP-dependent protease HslV (EC 3.4.25.-) | Q8ZJJ4 | 0.0732 |
| Outer membrane lipoprotein SmpA, a component of the essential YaeT outer-membrane protein assembly complex | Q7CH39 | -0.0116 |
| Polymyxin resistance protein PmrJ, predicted deformylase | Q7CIT9 | -0.353 |
| Fimbrial Z protein probable signal transducer fimZ | Q9ZC35 | -0.7165 |
| Sulfite reductase [NADPH] flavoprotein alpha-component (EC 1.8.1.2) | Q8ZBN6 | -1.0929 |
| Attachment invasion locus protein precursor | Q0WCZ9 | -1.14 |

*The LRC also includes an intercept term of 0.2996and optimal tuning parameters of $\lambda=$0.0209 and $\tau=0.5$. See Methods.

^Ϯ^ Shaded cells indicate proteins whose abundance changed significantly between the ancestor wild isolates and the descendant lineages in our previous study (Leiser ref)

^ϮϮ^ Although this polypeptide was not identified by Leiser et al as significantly changing, the B subunit of the same protein was identified.
