# Supplementary material for: Protein abundances can distinguish between naturally-occurring and laboratory strains of Yersinia pestis, the causative agent of plague
Source: PLoS One. 2017 Aug 30;12(8):e0183478. doi: 10.1371/journal.pone.0183478 (PMC5576697; doi:10.1371/journal.pone.0183478)
Supplement: S2 Table — (DOCX) [file pone.0183478.s006.docx]

**Table S2. Protein presence/absence features selected to distinguish analytical facility**

| **Protein name** | **Uniprot id** | **Coefficient** |
| --- | --- | --- |
| Nitrogen regulation protein NR(I) | Q0WKS0 | 0.2923 |
| Putative exported protein | Q7CLC2 | 0.6786 |
| Putative exported protein | Q0WKJ6 | 0.7645 |
| ATP-dependent protease HslV (EC 3.4.25.-) | Q8ZJJ4 | 0.0147 |
| LSU ribosomal protein L23p (L23Ae) | P69963 | 0.0221 |
| Putative primase, phage-associated | Q74QA8 | -0.3013 |
| 3-hydroxyacyl-[acyl-carrier-protein] dehydratase FabZ | Q8ZH57 | 1.0504 |
| Deoxycytidine triphosphate deaminase (EC 3.5.4.13) | Q8ZFZ8 | 0.509 |
| Major sodium/proline symporter | Q7CI08 | 0.3981 |
| Putative short chain dehydrogenase | Q0WEV2 | 0.2864 |
| 1,4-dihydroxy-2-naphthoyl-CoA synthase | Q74T55 | 1.8608 |
| Acetate kinase (EC 2.7.2.1) | Q8ZDJ6 | 0.2878 |
| Putative magnesium and cobalt efflux protein corC | Q7CJU8 | 0.1044 |
| L-proline glycine betaine binding periplasmic protein | Q7CJT4 | 0.7503 |
| Putative lipoprotein | Q0WCZ2 | 0.2981 |
| Xanthine-guanine phosphoribosyltransferase (EC 2.4.2.22) | Q8ZC05 | 0.4631 |
| GMP reductase (EC 1.7.1.7) | Q8ZBI2 | 0.865 |
| Glyoxylate/hydroxypyruvate reductase B | Q0W9V5 | 0.1885 |
