# Supplementary material for: Protein abundances can distinguish between naturally-occurring and laboratory strains of Yersinia pestis, the causative agent of plague
Source: PLoS One. 2017 Aug 30;12(8):e0183478. doi: 10.1371/journal.pone.0183478 (PMC5576697; doi:10.1371/journal.pone.0183478)
Supplement: S3 Table — (DOCX) [file pone.0183478.s007.docx]

**TABLE S3. Protein Identifiers Selected to Classify Culture Medium Using Relative Abundance Data**

| **Protein name** | **Gene locus** | **Uniprot identifier** | **Coefficients for different media*** | | | | |
| --- | --- | --- | --- | --- | --- | --- | --- |
|  |  |  | **BCS** | **BHI** | **DMEM** | **LB** | **TSB** |
| Pesticin | YPPCP1.05c | Q57159 | -0.5171 | 3.3030 | -4.0734 | 1.3073 | -0.0197 |
| Protease VII (Omptin) precursor (EC 3.4.23.49) pla | YPPCP1.07 | P17811 | -0.3438 | -0.2009 | 0.3134 | 0.0908 | 0.1405 |
| Aspartate--ammonia ligase (EC 6.3.1.1) | YPO0003 | Q8ZJT3 | -0.0032 | -0.0020 | 0.0031 | 0.0019 | 0.0002 |
| Transcriptional activator NhaR | YPO0471 | Q7CG78 | 0.7986 | 0.7507 | 0.7249 | -0.6642 | -1.6099 |
| FIG01220011: hypothetical protein | YPO0625 | Q7CGF6 | -0.2226 | 0.9618 | -0.2850 | -0.0761 | -0.3781 |
| Putative translational inhibitor protein | YPO0627 | Q7CGF7 | 0.0723 | -0.1721 | 0.1107 | 0.1236 | -0.1345 |
| Hypothetical flavoprotein | YPO1039 | Q7CH13 | 0.1886 | -0.5094 | 0.4169 | 0.3984 | -0.4945 |
| Outer membrane protein assembly factor BamE | YPO1104 | Q7CH39 | 0.0567 | 0.0930 | 0.1211 | -0.1173 | -0.1535 |
| Galactokinase (EC 2.7.1.6) | YPO1137 | Q8ZGY3 | 0.0044 | -0.0115 | -0.0067 | 0.0109 | 0.0030 |
| UDP-glucose 4-epimerase (EC 5.1.3.2) | YPO1139 | Q9F7D4 | -0.1270 | -0.0567 | 0.0812 | 0.2019 | -0.0994 |
| Putative periplasmic substrate-binding transport protein | YPO1310 | Q0WHA3 | -0.0125 | -0.0089 | 0.0130 | 0.0200 | -0.0116 |
| Translation initiation factor 1 | YPO1370 | P65115 | -1.1668 | -0.9689 | 0.8977 | 0.2010 | 1.0371 |
| Seryl-tRNA synthetase (EC 6.1.1.11) | YPO1379 | Q8ZGC4 | 0.5115 | -0.6174 | 0.4412 | -0.4083 | 0.0730 |
| 3-hydroxydecanoyl-[acyl-carrier-protein] dehydratase (EC 4.2.1.60) | YPO1430 | Q8ZG80 | 0.6089 | 0.7837 | 0.4751 | -1.4357 | -0.4321 |
| LSU ribosomal protein L32p | YPO1595 | Q8ZFT9 | 0.0391 | -0.0004 | 0.0288 | -0.1522 | 0.0847 |
| Uncharacterized protein | YPO1864 | Q74V09 | 0.2886 | -0.1895 | 0.2199 | -0.1211 | -0.1979 |
| iron aquisition 2,3-dihydroxybenzoate-AMP ligase (EC 2.7.7.58,Irp5) | YPO1907 | Q56950 | 0.5157 | -2.0445 | 0.6158 | 0.8123 | 0.1007 |
| iron aquisition yersiniabactin synthesis enzyme salicyl-AMP ligase (Irp1,polyketide synthetase) | YPO1911 | Q7CI41 | -0.1069 | 0.0719 | 0.1169 | -0.1055 | 0.0236 |
| Putative lipoprotein yceB precursor | YPO2039 | Q0WFB7 | -1.0652 | 0.5744 | 1.6998 | -0.8851 | -0.3238 |
| Zinc ABC transporter, periplasmic-binding protein ZnuA | YPO2061 | Q8ZEU2 | -0.2361 | -0.2740 | 0.4417 | 0.4034 | -0.3350 |
| NADH-ubiquinone oxidoreductase chain B (EC 1.6.5.3) | YPO2554 | Q7CJ93 | 0.3293 | -0.2740 | 0.1841 | -0.0092 | -0.2302 |
| Urease gamma subunit (EC 3.5.1.5) | YPO2665 | P69994 | 0.0919 | 0.2840 | 0.0948 | -0.1450 | -0.3257 |
| Putative long-chain fatty acid transport protein | YPO2744 | Q74SY3 | -1.2206 | 0.4815 | 0.6782 | -0.3471 | 0.4081 |
| Phosphoribosylaminoimidazole carboxylase ATPase subunit (EC 4.1.1.21) | YPO3077 | Q7CJZ2 | 0.1519 | -0.6230 | 0.1633 | 0.4904 | -0.1826 |
| Thiamin biosynthesis lipoprotein ApbE | YPO3234 | Q0WC52 | 0.8254 | -1.5821 | 0.5900 | -1.1839 | 1.3507 |
| Putative 2-hydroxy-3-oxopropionate reductase (EC 1.1.1.60) | YPO3648 | Q7CL58 | -0.0336 | -0.0854 | 0.0204 | 0.0575 | 0.0411 |
| Maltoporin (maltose/maltodextrin high-affinity receptor, phage lambda receptor protein) | YPO3711 | Q8ZAS9 | 0.4620 | -1.0490 | 0.5086 | 0.4224 | -0.3439 |
| Bifunctional purine biosynthesis protein PurH | YPO3728 | Q8ZAR3 | -0.1822 | -0.2234 | 0.1993 | 0.2587 | -0.0524 |
| Putative iron transport protein | YPO4022 | Q0WA00 | 0.3911 | -0.6112 | 0.1872 | -1.0293 | 1.0622 |

*BCS: Best Case Scenario medium; BHI: Brain-heart infusion; DMEM: Dulbecco’s Modified Eagle’s Medium; LB: Luria-Bertani broth; TSB: Tryptic soy broth
