# Supplementary material for: Protein abundances can distinguish between naturally-occurring and laboratory strains of Yersinia pestis, the causative agent of plague
Source: PLoS One. 2017 Aug 30;12(8):e0183478. doi: 10.1371/journal.pone.0183478 (PMC5576697; doi:10.1371/journal.pone.0183478)
Supplement: S4 Table — (DOCX) [file pone.0183478.s008.docx]

**TABLE S4. Protein Identifiers Selected to Classify Culture Medium Using Presence/Absence Data**

| **Protein name** | **Gene locus** | **Uniprot identifier** | **Coefficients for different media*** | | | | |
| --- | --- | --- | --- | --- | --- | --- | --- |
|  |  |  | **BCS** | **BHI** | **DMEM** | **LB** | **TSB** |
| Virulence-associated V antigen lcrV | YPCD1.31c | P0C7U7 | 0.5950 | -0.4878 | 0.6064 | -0.2863 | -0.4272 |
| Type III secretion outermembrane negative regulator of secretion (TyeA) | YPCD1.38c | P69968 | 0.0000 | 0.0000 | 0.0000 | 0.0000 | 0.0000 |
| Yop proteins translocation protein Q | YPCD1.43 | P42713 | 0.1489 | -0.2817 | 0.1879 | -0.2225 | 0.1673 |
| Aspartate--ammonia ligase (EC 6.3.1.1) | YPO0003 | Q8ZJT3 | 0.2627 | 0.3011 | 0.2878 | -0.2545 | -0.5970 |
| Formamidopyrimidine-DNA glycosylase (EC 3.2.2.23) | YPO0052 | Q8ZJP0 | -0.4539 | -0.4730 | 0.8458 | 0.6117 | -0.5306 |
| Putative membrane transport protein | YPO0363 | Q7CKM5 | 0.0173 | -0.0236 | 0.2028 | 0.2766 | -0.4731 |
| Transcriptional activator NhaR | YPO0471 | Q7CG78 | -0.2328 | 0.9186 | -0.2173 | -0.2103 | -0.2583 |
| ABC-type cobalamin/Fe3+-siderophores transport systems, periplasmic components | YPO0955 | Q74QI3 | -0.0705 | -0.0422 | 0.0948 | -0.0288 | 0.0467 |
| Ferrichrome-iron receptor | YPO0956 | Q0WI86 | -0.0598 | -0.0426 | 0.0909 | -0.0350 | 0.0466 |
| Hypothetical flavoprotein | YPO1039 | Q7CH13 | 0.3714 | 0.3722 | 0.4208 | -0.4941 | -0.6702 |
| FIG00905232: hypothetical protein | YPO1064a | Q8ZH47 | -0.2463 | -0.2862 | 0.4914 | -0.0093 | 0.0504 |
| Putative inner membrane protein, TolR protein | YPO1122 | Q7CH54 | -0.0225 | -0.0282 | 0.0239 | 0.0130 | 0.0138 |
| Galactokinase (EC 2.7.1.6) | YPO1137 | Q8ZGY3 | -0.1183 | -0.1137 | 0.1380 | 0.1566 | -0.0626 |
| Putative periplasmic substrate-binding transport protein | YPO1310 | Q0WHA3 | -0.0439 | -0.0462 | 0.0343 | 0.0150 | 0.0408 |
| Glutaredoxin 1 | YPO1327 | Q7CHG3 | 0.0545 | 0.0837 | 0.0616 | -0.1410 | -0.0588 |
| Putative periplasmic substrate-binding transport protein | YPO1343 | Q7CHH1 | 1.3169 | -0.3868 | -0.5024 | -0.1437 | -0.2840 |
| Translation initiation factor 1 | YPO1370 | P65115 | 0.0473 | -0.0592 | 0.0493 | -0.0534 | 0.0159 |
| Seryl-tRNA synthetase (EC 6.1.1.11) | YPO1379 | Q8ZGC4 | 0.0733 | 0.1934 | 0.0824 | -0.3679 | 0.0188 |
| Quinone oxidoreductase (EC 1.6.5.5) | YPO1653 | Q0WGC5 | 0.0377 | -0.0749 | 0.0406 | -0.0969 | 0.0935 |
| Uncharacterized protein | YPO1864 | Q74V09 | 0.3401 | -1.3706 | 0.2872 | 0.4096 | 0.3336 |
| iron aquisition yersiniabactin synthesis enzyme (Irp1,polyketide synthetase) | YPO1911 | Q7CI41 | -0.1918 | 0.2009 | 0.2822 | -0.2088 | -0.0825 |
| Anthranilate synthase, aminase component (EC 4.1.3.27) | YPO1916 | Q9Z396 | -0.0138 | -0.0017 | 0.0208 | -0.0244 | 0.0191 |
| Phage shock protein B | YPO2350 | Q7CIQ7 | 0.0157 | -0.0591 | 0.0413 | 0.0247 | -0.0226 |
| Urease gamma subunit (EC 3.5.1.5) | YPO2665 | P69994 | -0.2501 | 0.1766 | 0.1421 | -0.1072 | 0.0386 |
| Phosphoribosylaminoimidazole carboxylase ATPase subunit (EC 4.1.1.21) | YPO3077 | Q7CJZ2 | 0.4059 | -0.7328 | 0.4610 | -0.8220 | 0.6880 |
| Peptide methionine sulfoxide reductase MsrA (EC 1.8.4.11) | YPO3525 | Q8ZB94 | -0.0133 | 0.0400 | 0.0377 | -0.0976 | 0.0331 |
| Penicillin-binding protein activator LpoA | YPO3548 | Q0WBA3 | 0.0562 | -0.5876 | 0.1398 | 0.1337 | 0.2579 |
| D-ribulokinase (EC 2.7.1.47) | YPO3637 | Q0WB16 | 0.0085 | -0.0860 | 0.0618 | 0.0665 | -0.0508 |
| TldE protein, part of TldE/TldD proteolytic complex | YPO3692 | Q7CL78 | 0.0159 | 0.0651 | 0.0488 | -0.1570 | 0.0271 |
| Maltoporin (maltose/maltodextrin high-affinity receptor, phage lambda receptor protein) | YPO3711 | Q8ZAS9 | -0.0334 | -0.0435 | 0.0451 | 0.0409 | -0.0091 |
| Putative receptor | YPO3817 | Q7CKX1 | 0.0970 | -0.0439 | 0.0144 | 0.1467 | -0.2142 |
| Putative lipoprotein | YPO3846 | Q7CKY6 | -0.0548 | -0.0799 | 0.1235 | 0.0243 | -0.0131 |
| Acetylornithine deacetylase (EC 3.5.1.16) | YPO3928 | Q8ZA85 | 0.1010 | 0.2229 | 0.1178 | -0.3073 | -0.1345 |
| PTS system mannitol-specific enzyme IIABC components | YPO4068 | Q7CFP7 | 0.0869 | -0.2316 | 0.0753 | 0.0736 | -0.0042 |
| Putative periplasmic solute-binding protein | YPO4111 | Q7CFN1 | -0.3059 | 0.0854 | 0.1394 | -0.0467 | 0.1278 |

*BCS: Best Case Scenario medium; BHI: Brain-heart infusion; DMEM: Dulbecco’s Modified Eagle’s Medium; LB: Luria-Bertani broth; TSB: Tryptic soy broth
